# *Mycobacterium florentinum* pulmonary disease: a case report and review of the literature

**DOI:** 10.1186/s12879-025-12321-3

**Published:** 2025-12-17

**Authors:** Fabian Leo, Silke Polsfuss, Anne-Sophie Przewosnik, Christian Grohé, Christoph Lange

**Affiliations:** 1 Department of Respiratory Medicine, Evangelische Lungenklinik, Berlin, Germany; 2 Institute of Microbiology, Immunology and Laboratory Medicine, HELIOS Klinikum Emil-von-Behring, Berlin, Germany; 3 Division of Clinical Infectious Diseases, Research Centre Borstel, Leibniz Lung Center, Borstel, Germany; 4 German Centre for Infection Research (DZIF), Partner Site Hamburg-Lubeck-Borstel-Riems, Borstel, Germany; 5 Respiratory Medicine and International Health, University of Lübeck, Lübeck, Germany; 6 Division of Global Health, Baylor College of Medicine and Texas Children´s Hospital, Houston, TX USA

**Keywords:** Nontuberculous mycobacteria, *Mycobacterium florentinum*, Pulmonary aspergillosis, *Aspergillus fumigatus*, Amikacin, Inhalation drug administration

## Abstract

**Background:**

Following the initial description of *Mycobacterium florentinum* in 2005, very few clinical cases have been reported and the optimal antimicrobial treatment and clinical outcomes are uncertain. Amikacin liposome inhalation suspension (ALIS) has received approval for the treatment of *Mycobacterium avium/intracellulare complex* (MAC) pulmonary disease. However, there is little experience with its use for infections caused by less common nontuberculous mycobacteria (NTM). Moreover, the awareness of uncommon adverse effects is still limited.

**Case presentation:**

A 69-year-old female patient suffering from nodular bronchiectatic *Mycobacterium florentinum* pulmonary disease was treated with azithromycin, ethambutol, and rifampicin, administered three times per week. After 13 months, owing to treatment failure, the therapy was changed to ALIS, moxifloxacin, and clofazimine. This resulted in rapid and sustained culture conversion. Concurrently, the patient exhibited increased cough and sputum, which was consistent with a clinical diagnosis of aspergillosis, as confirmed by evidence of *Aspergillus fumigatus* in respiratory specimens and a significant increase in serum *Aspergillus* IgG antibody levels. Following a six-month course of antifungal therapy, a marked improvement in the patient’s symptoms was observed.

**Conclusions:**

As with MAC pulmonary disease, combination antimicrobial therapy including ALIS was successful in a patient affected by a difficult-to-treat pulmonary infection caused by *Mycobacterium florentinum,* a rare NTM pathogen. Combination antibiotic treatment including ALIS may also be considered for other difficult-to-treat, non-MAC NTM- pulmonary diseases. During treatment, patients should be monitored for the emergence of Aspergillus co-infection.

**Supplementary Information:**

The online version contains supplementary material available at 10.1186/s12879-025-12321-3.

## Background

*Mycobacterium florentinum* was first characterized by Tortoli et al. in 2005 in a case series comprising eight isolates from human samples. It is closely related to *M. triplex*, *M. lentiflavum* and *M. montefiorense*, but is clearly distinguishable by unique 16S rRNA and internal transcribed spacer (ITS) sequences as well as a unique PCR restriction analysis (PRA) pattern of the 65 kDa heat-shock protein-encoding gene (hsp65) [1]. More than half of the isolates were considered clinically relevant infections (four out of six from respiratory samples, and one from cervical lymph node tissue). However, treatment and clinical outcome data were not reported. The authors also identified a previously documented case of NTM lung disease from 2001 as being attributable to a *Mycobacterium florentinum* infection [1, 2]. The patient was a 67-year-old female from Finland who was diagnosed with nontuberculous mycobacterial pulmonary disease (NTM-PD) with a nodular bronchiectatic radiologic phenotype. Data from antimicrobial susceptibility testing (AST) were not reported. However, the patient was considered clinically cured after 18 months of treatment with clarithromycin, ethambutol, rifampicin and ciprofloxacin. In the following years, only two additional case reports of *M. florentinum* infection have been published in the English-language literature: one in 2010, which detailed a second case of cervical lymphadenitis in a child, and one in 2014, documenting a single case of synovitis in an immunocompromised individual [3, 4]. In the case of cervical lymphadenitis, therapy consisted of complete surgical lymph node excision and no relapse occurred within 12 months of follow-up observation [3]. The authors of this report also conducted a data query at a national reference laboratory in the United States (Associated Regional and University Pathologists Laboratories), covering the period between 2006 and 2010. This revealed four additional documented *M. florentinum* isolates: a 76-year-old man (source: bronchial aspirate), a 47-year-old man (sputum), a 5-year-old girl (neck lymph node), and a 46-year-old man (unspecified source). The cases were submitted from different regions in the United States. No information was provided on the clinical relevance of these findings [3]. Nukui et al. described a case of synovitis in a 65-year-old, immunocompromised patient with a history of systemic lupus erythematosus (SLE), chronic kidney disease and long-term prednisolone therapy. *M. florentinum* was found to be the causative pathogen, and treatment consisted of surgical debridement and antimycobacterial drugs (clarithromycin, rifampicin, and levofloxacin). The patient died unexpectedly after three months from acute aortic dissection [4]. A synopsis of all cases published to date, with at least basic clinical data and available AST results, is shown in Table 1. Due to the limited number of cases reported thus far, the most effective treatment regimen remains unclear.

**Table 1:** Overview of clinical Mycobacterium florentinum cases in the English-language literature

Reference/ year	Gender and age (years)	Source	Clinical presentation	CS	MIC AMK mg/l	MIC CIP mg/l	MIC CLR mg/l	MIC CFZ mg/l	MIC EMB mg/l	MIC MOX mg/l	MIC LFX mg/l	MIC RFB mg/l	MIC RMP mg/l
[1]/2005	F, 6	Lymph node	Lymphadenitis	yes	2	4	2	0.12	8	n/a	n/a	0.25	16
	F, 82	Sputum	Emphysema	no	0.5	128	2	0.12	4	n/a	n/a	0.06	8
	M, 33	Stools	Weight loss	no	4	128	2	0.5	8	n/a	n/a	0.25	4
	F, 70	Sputum	Pulmonary fibrosis, bronchiectasis	yes	n/a								
	F, 93	Sputum, gastric aspirate	Hemoptysis, fever	n/a									
	F, 84	Sputum	Hemoptysis, pleurisy	no									
	M, 65	Sputum	Pneumonia	yes									
	F, 64	Sputum	Pneumonia	yes									
[2]/2001	F, 67	Sputum, BAL	Hemoptysis	yes									
[3]/2010	F, 3	Lymph Node	Lymphadenitis	yes	0.5	8	0.5	n/a	4	1	n/a	n/a	0.12
[4]/2014	F, 65	Synovia (wrist)	Arthritis	yes	16	n/a	2	n/a	8	n/a	16	n/a	4
Leo et al. 2025 †; ††	F, 69	Sputum	Pneumonia, hemoptysis	yes	16	> 16	1	n/a	n/a	> 8	n/a	0.25	> 8

Basic demographic data, isolation source, clinical presentation, clinical significance and antimicrobial susceptibility testing results of M. florentinum cases reported in the literature, including this case report. F= female; M = male; CS = clinical significance; MIC = minimal inhibitory concentration; AMK = Amikacin, CIP = Ciprofloxacin; CLR = Clarithromycin; CFZ = Clofazimine; EMB = Ethambutol; MOX = Moxifloxacin; LFX = Levofloxacin; RFB = Rifabutin; RMP = Rifampicin; n/a: not applicable (not done)

† Antimicrobial susceptibility testing (AST) was performed and assessed by broth microdilution method (SensititreTM Myco susceptibility plates SLOMYCOI, Thermo Fisher Diagnostics GmbH, Germany) according to Clinical and Laboratory Standards Institute (CLSI M24Ed2 and M62Ed1 (November 2018 and CLSI M24S Ed2 (February 2023) guideline

†† More comprehensive data including repeat AST of isolates during antimycobacterial treatment can be found in Additional file 2 (Supplemental information)

Amikacin liposome inhalation suspension (ALIS) is currently approved for the treatment of pulmonary infection caused by nontuberculous mycobacteria belonging to the *Mycobacterium avium*/*intracellulare* complex (MAC). There is little experience with the use of ALIS in less frequent NTM species. The use of systemic or inhaled antibiotics has been related to an increase in airway colonization with *Aspergillus spp*. in patients with cystic fibrosis (CF), non-CF bronchiectasis and NTM-PD [5–8]. In patients with NTM-PD, the development of chronic pulmonary aspergillosis (CPA), especially the cavitary type (CCPA), is a prognostic factor for mortality [9]. A synopsis of CPA subtypes, as defined by the European Society of Clinical Clinical Microbiology (ESCMID) and European respiratory Society (ERS) [10] is presented in Additional File 1. Owing to the many common symptoms and radiological features of NTM-PD and CPA, distinguishing between colonization and clinically relevant aspergillosis may be difficult. The specific impact of ALIS on *Aspergillus spp.* colonization and development of CPA has not been studied [11].

The objective of this report is to describe a very rare case of *M. florentinum* pulmonary disease with a view to its management utilizing an ALIS-based antibiotic regimen and close attention to possible adverse effects, including the emergence of fungal coinfection.

## Case presentation

### NTM-PD diagnosis and clinical course prior to antimicrobial treatment

**Fig. 1:**
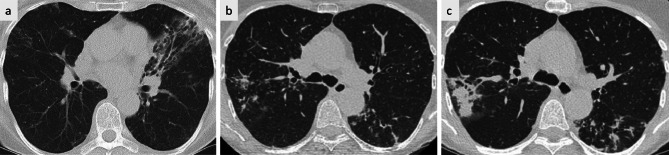
Thoracic CT scans (axial plane, 1-mm-slices). **a**: CT thorax scan 12/2018 revealing bronchiectasis predominantly in the left lung (Lingula). **b**: Nodules and consolidations consistent with progressive NTM pulmonary disease in 12/2020. **c**: Disease progression under macrolide-based antimicrobial regimen in 01/2022

In December 2018, a 69-year-old female patient (163 cm, 53 kg, BMI 19.9 m^2^/kg)) was first referred to our clinic for refractory pneumonia. She reported chronic cough and weight loss (6 kg in 12 months). The patient was a non-smoker and had no history of diabetes mellitus, autoimmune disease, malignancy, or other chronic disease except for gastro-esophageal reflux disease that had been treated with pantoprazole. She reported no current or past use of inhaled or systemic corticosteroids or other immunosuppressants. There was no history of environmental exposure to water-related areas, soil, hot springs or spas. Chest computed tomography (CT) revealed bronchiectasis and peribronchial consolidation focused in the middle lobe, lingula, and left lower lobe (Fig. 1a). C-reactive protein, blood leucocytes, lymphocytes, monocytes, eosinophils, alpha1-antitrypsin and immunoglobulins IgA, IgE, IgG and IgM were within the respective reference ranges, whereas *Aspergillus* IgG antibodies were marginally elevated (ELISA 63 U/ml, reference < 50 U/ml, Serion Diagnostics GmbH, Wuerzburg, Germany) and serum *Aspergillus* antigen (galactomannan) was negative (Index 0.09, reference < 0.5). (Bronchoalveolar lavage (BAL) revealed 95% neutrophils and evidence of *Staphylococcus aureus* (MSSA [10^2^/ml]), which was treated with ampicillin/sulbactam for 14 days. Acid fast bacilli (AFB) remained undetectable by microscopy of auramine-stained BAL fluid and in three sputum samples, whereas growth was detected in liquid culture media (MGIT™, Becton Dickinson, Sparks, Md., USA) from BAL fluid and two sputum samples. The time-to-positivity of the first culture was 15 days. Subsequent amplification and sanger sequencing of the 16S rDNA gene and internal transcribed spacer (ITS) identified *M. florentinum* by NCBI Blast (https://blast.ncbi.nlm.nih.gov/Blast.cgi): both sequences showed 100% identity with the signature sequences published by Tortoli et al. for the initial description of the species *M. florentinum* (GenBank/EMBL/DDBJ accession number of 16S rRNA gene and ITS region sequence of strain FI-93171^T^ is AJ616230) [1]. In 02/2019, symptoms and radiologic findings improved, and the patient was managed with sodium saline inhalation and physical therapy using a Flutter™ device. For a 24- month period of “watchful waiting”, specific antimycobacterial treatment was withheld according to the patient’s informed preference, although sputum cultures grew *M. florentinum* again during this period (in 12/2019).

### Disease progression and first-line (macrolide-based) antimicrobial treatment

In 12/2020, the patient reported frequent hemoptysis, and CT imaging showed increasing nodules and consolidations in the right upper lobe, middle lobe and left lower lobe (Fig. 1b). Sputum cultures grew *Mycobacterium avium* once. This isolate was tested susceptible to clarithromycin, as was the previously isolated *M. florentinum*. In 02/2021, antimycobacterial treatment was started, using a regimen of azithromycin (AZM), ethambutol (EMB) and rifampicin (RMP) three times weekly. In 04/2021, sputum culture for mycobacteria was negative, but became positive again in 06/2021 and 08/2021, with molecular species differentiation revealing *M. florentinum* on both occasions, but not *M. avium*. Treatment was continued because it was deemed effective against *M. avium*, the patient had less respiratory symptoms and there was no radiological progression. However, sputum microscopy detected AFB for the first time in 10/2021, indicating a high bacterial burden, and cultures grew *M. florentinum* again. Repeat in- vitro drug susceptibility testing excluded the development of macrolide resistance. The minimal inhibitory concentration (MIC) for Amikacin (AMK) was 16 mg/l, which corresponds to susceptibility according to the Clinical and Laboratory Standards Institute (CLSI) definition (see Additional file 2). *Aspergillus* IgG antibodies were measured repeatedly and showed an increase compared to the baseline value, but without any change over time (04/2021: 120 U/ml, 09/2021: 117 U/ml, 02/2022: 113 U/ml). Serum galactomannan levels remained normal throughout the treatment course (04/2021: Index 0.13, 09/2021: Index 0.03, 02/2022: Index 0.12).

### Switch to ALIS-based treatment, clinical course and outcome

**Fig. 2:**
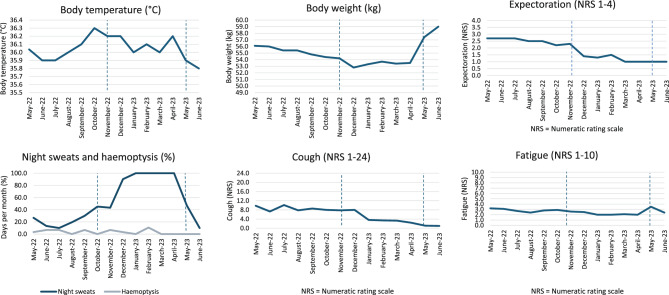
Time course of symptoms under treatment (antimycobacterial therapy from March 2022 until April 2023; antifungal therapy from November 2022 until May 2023). Symptoms were recorded by the patient on a daily basis (beginning in May 2022), using the “NTM clinical Diary”, developed at the German center for infection Research (DZIF). Mean values for each month were calculated from the daily entries for body temperature, body weight and NRS values describing the extent of cough, expectoration and fatigue. Night sweats and hemoptysis are presented as percentages of days per month on which they occurred

**Fig. 3:**
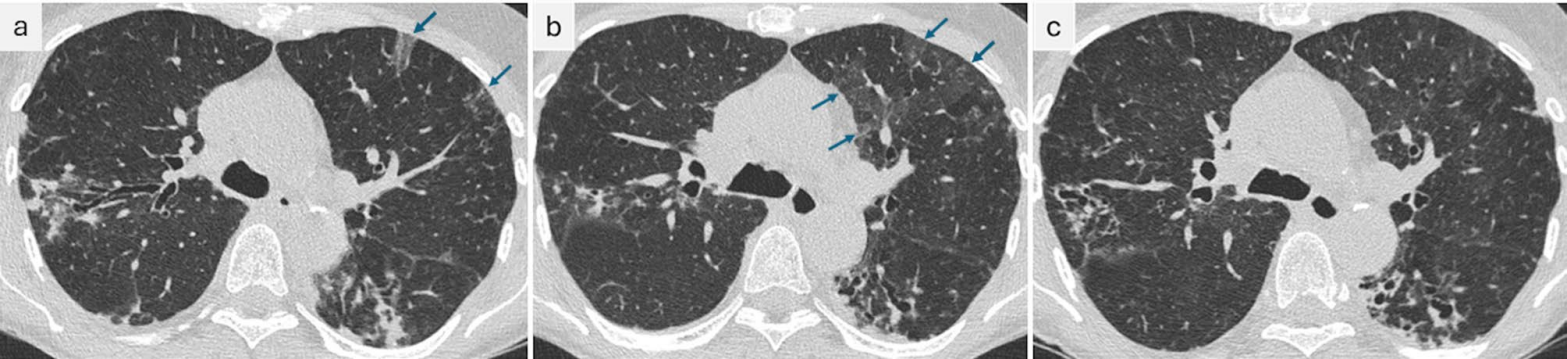
Thoracic CT scans (axial plane, 1-mm-slices). **a**. CT scan at 3 months of ALIS-based antimycobacterial treatment in 06/2022: decreasing consolidations compared to Fig. 1c. Focal ground glass opacities (GGO) in the left upper lobe (arrows). **b**. CT scan after completion of antimicrobial treatment in 05/2023. Increased GGO in the left upper lobe (arrows) **c**. Decrease of GGO at 7 months after completion of ALIS treatment

In 01/2022, CT imaging revealed progressive consolidation in the right upper lobe (segment 2) and progressive bronchiolitis in the inferior lower lobes and middle lobe (Fig. 1c). In 03/2022 ( = month 0), the treatment was switched to ALIS 590 mg/d by inhalation, clofazimine 100 mg/d orally and moxifloxacin 400 mg/d orally. With this regimen, culture conversion was documented in 04/2022 (month +1), and follow-up CT showed improvement in 06/2022 (month +3, Fig. 3a). In 09/2022 (month +6), the patient described an episode of increasing cough and sputum, while CT showed a mixed response, and mycobacterial cultures remained negative. Proof of *A. fumigatus* in sputum samples and a marked rise in serum *Aspergillus* IgG antibodies (207 U/ml) prompted the initiation of antifungal treatment with oral voriconazole (VCZ) in 11/2022 (month +8). Therapeutic drug monitoring was performed during treatment, and all VCZ trough levels were within the therapeutic range (1–5 mg/l). The patient´s clinical status improved gradually, as indicated by changes in body weight, cough, fatigue, and other factors (Fig. 2). Nonetheless, night sweats persisted for a period of several months and were considered to be potentially unrelated to the underlying disease. Antimycobacterial treatment (ALIS, clofazimine, and moxifloxacin) was terminated in 04/2023 (month +13; 12 months after sputum conversion), and VCZ was continued until 05/2023 (treatment duration of 6 months). Chest CT at the end of treatment showed radiological improvement (Fig. 3b). During follow-up, sustained sputum conversion was documented until the last contact with the patient in 03/2025 (month +36, 23 months after the end of treatment). The level of *Aspergillus* IgG antibodies decreased to 138 U/ml at the conclusion of VCZ treatment and to 98 U/ml at six months thereafter. Twenty-two months after completing VCZ treatment, the level *Aspergillus* IgG was < 50 U/ml. Follow-up sputum samples exploring respiratory pathogens other than mycobacteria revealed *S. aureus*, *Enterobacteriaceae* and *Scedosporium spp*. and two episodes of antibiotic treatment for bronchiectasis exacerbation were necessary during nearly two years of follow-up.

### Adverse events during ALIS treatment

From the beginning of ALIS therapy, the patient reported variable dry cough and dysphonia related to inhalation. These symptoms were tolerable throughout the entire treatment course, without the need for discontinuation of the inhalations. Chest CT imaging at month +3 revealed focal ground glass opacities (GGO) in the upper lobes bilaterally and in segments 6 and 9 of the right lung (Fig. 3a). At month +6, the GGO decreased slightly, but at month +12, new GGO emerged in the right upper lobe and left upper and lower lobes (Fig. 3b). At month +20, seven months after the end of ALIS treatment, the extent of GGO was markedly decreased (Fig. 3c).

### Data query

A data query at the German National Reference Centre for Mycobacteria (Research Center Borstel) was conducted. No other strains of *M. florentinum* were identified during the period from January 2018 to April 2025.

## Discussion and conclusions

This case of *M. florentinum* pulmonary disease (*M. florentinum*-PD) is only the second documented case that provides detailed data on treatment and clinical outcomes. The other, by Suomalainen et. al in 2001, predates the classification of *M. florentinum* as an independent species. Initially, it was considered a subspecies of the closely related *M. triplex* taxon. A substantial case series pertaining to *M. triplex* pulmonary disease from Australia was published in 2019 [12]. The collection was composed of 39 cases, with a significant portion (approximately 45%) meeting the criteria for NTM-PD [13]. The AST results obtained for 14 isolates revealed that 93% were susceptible to macrolides. However, only six patients with pulmonary infection received antimicrobial therapy (including azithromycin or clarithromycin), with five patients demonstrating treatment success [12]. In conjunction with the existing data on AST for *M. florentinum* (Table 1), these findings support the selection of a macrolide-based combination as an initial therapy for the treatment of *M. florentinum* pulmonary disease.

ALIS has the potential to serve as an ancillary option for patients who are difficult to treat, e.g. due to poor tolerability of oral drugs, or in cases where first-line therapy has failed. In addition to its on-label use for MAC-PD, ALIS was reported to be effective in CF bronchiectasis and non-CF bronchiectasis patients with *M. abscessus* infection [14]. In the case of *M. florentinum*-PD presented here, an ALIS-based treatment resulted in rapid culture conversion and microbiological and clinical cure as defined by the NTMnet group consensus [15].

Remarkably, the MIC for amikacin was higher in this case than in the other *M. florentinum* strains reported in the literature. However, compared with conventional parenteral or inhaled amikacin, the use of ALIS results in higher concentrations of amikacin in macrophages, lung tissue and biofilms, potentially enhancing its efficacy against mycobacteria [16]. Furthermore, in-vitro data across different NTM species suggest antimycobacterial synergy between amikacin and clofazimine, which was also part of the treatment regimen [17].

Moxifloxacin was selected as an additional drug because it has been used successfully as part of multidrug combination therapies in the treatment of pulmonary infections caused by M. triplex [12]. When treatment success became evident, moxifloxacin was maintained within the treatment regimen, despite a high MIC of > 8 mg/L.

Inhaled antibiotics may promote airway colonization by *A. fumigatus* and other fungi. Insights are available from studies of CF -bronchiectasis: Microbiological data from two randomized, controlled trials studying inhaled tobramycin vs. placebo showed that isolation of *Aspergillus* and *Candida* was increased in the tobramycin group. However, the detection of fungal pathogens had no significant influence on the FEV1 improvement achieved through tobramycin inhalation. No treatment of either fungal pathogen was initiated during the study period [5]. In a more recent cohort of CF patients from Sweden, the use of inhaled antibiotics was also associated with *A. fumigatus* colonization (adjusted OR 3.1, 95% CI 1.6–5.9, *p* < 0.05) but not with adverse effects on lung function [6]

In general, the association between *Aspergillus* infection and NTM-PD has long been known, but it is not clear whether NTM-PD predisposes individuals to *Aspergillus* infection or vice-versa [18]. Raats et al. studied the occurrence of *Aspergillus* isolation in a cohort of patients diagnosed with NTM-PD. During a median follow-up of 46 months after NTM-PD diagnosis, *Aspergillus* was isolated from at least one respiratory sample in 130/497 patients (26.2%). Inhaled corticosteroid use, a nodular bronchiectatic CT pattern and NTM-PD treatment initiation were more common in patients with isolated *Aspergillus* than in those without (p-values of 0.01, 0.03 and < 0.001, respectively). In patients who were treated for NTM-PD, most of the isolates were first detected during or after antibiotic therapy (65/92; 70.7%). However, treatment outcomes were not different between the *Aspergillus* group and the non-*Aspergillus* group [8]. Although mere colonization does not appear to be critical, the development of CPA is a prognostic factor for mortality in patients with NTM-PD [19, 20]. Jhun et al. reported a hazard ratio (HR) of 3.49 (CI 2.07–5.88, *p* < 0.001) in a univariate regression model and an HR of 1.77 (1.01–3.11, *p* = 0.047) in a multivariate Cox proportional- hazards regression model [20]. The evolution from *Aspergillus* colonization to infection is poorly understood. Risk factors for developing CPA in NTM patients have been studied, but the influence of antimycobacterial treatment itself has not been investigated [9, 19]

In the case presented here, the patient did not meet the full diagnostic criteria for CPA, invasive aspergillosis (IA), or allergic bronchopulmonary aspergillosis (ABPA). However, the increase in *Aspergillu*s IgG levels was used as a surrogate marker of disease activity. The presence of an *Aspergillus* bronchitis phenotype of infection was considered, as described in the extant literature, primarily in patients with bronchiectasis [21]. Upon subsequent consideration, the clinical response to antifungal therapy (Fig. 2) supported this hypothesis. Moreover, a greater than 50% reduction in *Aspergillus* IgG was attained with antifungal treatment, a criterion for serologic improvement that was endorsed by the majority of experts in a consensus statement on definitions of treatment outcomes in CPA [22]

Hypersensitivity pneumonitis, or allergic alveolitis, has been described as adverse drug reaction in ALIS treatment in 1.8% of patients in the CONVERT open-label extension trial and in single case reports, prompting treatment discontinuation and/or glucocorticoid therapy [23–26]. In the case presented here, ground-glass opacities (GGO) – the radiological hallmark of hypersensitivity pneumonitis – may have been attributable to ALIS. However, GGO were transient and remained clinically inapparent. Therefore, no further investigations or treatment modifications were deemed necessary.

In summary, *Mycobacterium florentinum* is an exceedingly rare pathogen causing pulmonary disease in elderly patients and lymphadenitis in children, similar to other NTM species. Specific environmental or individual risk factors for *M. florentinum* infection are not known. A more comprehensive and advanced assessment of the hosts immune status in such rare infections should be considered, including evaluation of CD4-T-cell count, CD4/CD8 ratio, HIV antibody, and anti-Interferon-γ autoantibody testing. In the case of *M. florentinum-*PD presented here, a treatment regimen with ALIS, clofazimine and moxifloxacin was more effective than the combination of azithromycin, ethambutol and rifampicin three times weekly. The specific impact of ALIS treatment on *Aspergillus* colonization and infection remains to be clarified in future studies. Serial *Aspergillus* IgG antibodies should be determined prior to and during antimicrobial treatment for NTM-PD.

## Electronic supplementary material

Below is the link to the electronic supplementary material.

Supplementary Material 1

Supplementary Material 2

## Data Availability

The datasets supporting the conclusions of this article are included within the article and its additional files.

## Abbreviations

AFB: Acid-fast bacilli
ALIS: Amikacin liposomal inhalation suspension
AMK: Amikacin
AST: Antimicrobial susceptibility testing
AZM: Azithromycin
BMI: Body mass index
CF: Cystic fibrosis
CFZ: Clofazimine
CLSI: Clinical and Laboratory Standards Institute
CPA: Chronic pulmonary aspergillosis
CT: Computed tomography
EMB: Ethambutol
FEV1: Forced expiratory volume in one second
GGO: Ground-glass opacities
MAC: Mycobacterium avium/intracellulare complex
NTM: Nontuberculous mycobacteria
PCR: Polymerase chain reaction
VCZ: Voriconazole
