# Supplementary material for: Mycobacterium florentinum pulmonary disease: a case report and review of the literature
Source: BMC Infect Dis. 2025 Dec 17;26:103. doi: 10.1186/s12879-025-12321-3 (PMC12822083; doi:10.1186/s12879-025-12321-3)
Supplement: Supplementary file 1 — Supplementary Material 1 [file 12879_2025_12321_MOESM1_ESM.docx]

| Term | Definition |
| --- | --- |
| Simple aspergilloma | Single pulmonary cavity containing a fungal ball, with serological or  microbiological evidence implicating Aspergillus spp. in a  non-immunocompromised patient with minor or no symptoms and no  radiological progression over at least 3 months of observation. |
| CCPA | One or more pulmonary cavities (with either a thin or thick wall) possibly  containing one or more aspergillomas or irregular intraluminal material, with  serological or microbiological evidence implicating Aspergillus spp. with  significant pulmonary and/or systemic symptoms and overt radiological  progression (new cavities, increasing pericavitary infiltrates or increasing  fibrosis) over at least 3 months of observation. |
| CFPA | Severe fibrotic destruction of at least two lobes of lung complicating CCPA leading  to a major loss of lung function. Severe fibrotic destruction of one lobe with a  cavity is simply referred to as CCPA affecting that lobe. Usually the fibrosis is  manifest as consolidation, but large cavities with surrounding fibrosis may  be seen. |
| Aspergillus nodule | One or more nodules which may or may not cavitate are an unusual form of CPA.  They may mimic tuberculoma, carcinoma of the lung, coccidioidomycosis and  other diagnoses and can only be definitively diagnosed on histology. Tissue  invasion is not demonstrated, although necrosis is frequent. |
| SAIA | Invasive aspergillosis, usually in mildly immunocompromised patients, occurring  over 1–3 months, with variable radiological features including cavitation,  nodules, progressive consolidation with “abscess formation”. Biopsy shows  hyphae in invading lung tissue and microbiological investigations reflect those in  invasive aspergillosis, notably positive Aspergillus galactomannan antigen in  blood (or respiratory fluids). |

CCPA: chronic cavitary pulmonary aspergillosis; CFPA: chronic fibrosing pulmonary aspergillosis; SAIA: subacute invasive aspergillosis/chronic necrotizing/semi-invasive.

**Additional file 1:** Diagnostic criteria for different management of chronic pulmonary aspergillosis (CPA).Table modified from: Chronic pulmonary aspergillosis: rationale and clinical guidelines for diagnosis and management, Denning et al. European Respiratory Journal 2015 47(1): 45-68; DOI: https://doi.org/10.1183/13993003.00583-2015.
